# Are Pediatric Triage Systems Reliable in the Emergency Department?

**DOI:** 10.1155/2020/9825730

**Published:** 2020-07-10

**Authors:** Mohsen Ebrahimi, Amir Mirhaghi, Zohre Najafi, Hojjat Shafaee, Mahin Hamechizfahm Roudi

**Affiliations:** ^1^Department of Emergency Medicine, Faculty of Medicine, Mashhad University of Medical Sciences, Mashhad, Iran; ^2^Nursing and Midwifery Care Research Center, Mashhad University of Medical Sciences, Mashhad, Iran; ^3^Student Research Committee, School of Nursing and Midwifery, Shahid Beheshti University of Medical Sciences, Tehran, Iran; ^4^Department of Disaster Public Health, School of Public Health, Tehran University of Medical Sciences, Tehran, Iran

## Abstract

**Background:**

Few studies have focused on the agreement level of pediatric triage scales (PTSs). The aim of this meta-analytic review was to examine the level of inter-rater reliability of PTSs.

**Methods:**

Detailed searches of a number of electronic databases were performed up to 1 March 2019. Studies that reported sample sizes, reliability coefficients, and a comprehensive description of the assessment of the inter-rater reliability of PTSs were included. The articles were selected according to the COnsensus-based Standards for the selection of health status Measurement INstruments (COSMIN) taxonomy. Two reviewers were involved in the study selection, quality assessment, and data extraction and performed the review process. The effect size was estimated by *z*-transformation of reliability coefficients. Data were pooled with random-effects models, and a metaregression analysis was performed based on the method of moments estimator.

**Results:**

Thirteen studies were included. The pooled coefficient for the level of agreement was 0.727 (confidence interval (CI) 95%: 0.650–0.790). The level of agreement on PTSs was substantial, with a value of 0.25 (95% CI: 0.202–0.297) for the Australasian Triage Scale (ATS), 0.571 (95% CI: 0.372–0.720) for the Canadian Triage and Acuity Scale (CTAS), 0.810 (95% CI: 0.711–0.877) for the Emergency Severity Index (ESI), and 0.755 (95% CI: 0.522–0.883) for the Manchester Triage System (MTS).

**Conclusions:**

Overall, the reliability of pediatric triage systems was substantial, and this level of agreement should be considered acceptable for triage in the pediatric emergency department. Further studies on the level of agreement of pediatric triage systems are needed.

## 1. Introduction

Children increasingly visit emergency departments (ED) in the recent years [1]. Infections of the upper respiratory tract, asthma, and nonbacterial gastroenteritis were the most common diagnoses among frequent utilizers of the EDs [2]. However, children's complaints are not notably diverse in the ED. The increased number of children presenting to the EDs raises the importance of triaging them to the most appropriate level of care [3]. It becomes more of a priority due to the overcrowding of pediatric patients. In addition, nurses are less expert in triaging children in general hospitals compared with specialty pediatric hospitals [3].

Pediatric triage scales (PTSs) are used to prioritize children to receive care in the EDs [4]. They are essential to ensuring safety of patients and protection against undertriage [5]. Undertriage may result in serious delays in definitive care and an increased mortality or morbidity as well. PTSs must be reliable and correctly estimate the risk attributed to chief complaints and vital signs [6]. Inter-rater reliability is the most common method of reliability assessment in the triage room [7]. It represents the extent of agreement among clinicians. It is important to know to what extent triage scales are reliable because all triage scales were primarily developed for prioritizing adult patients and the pediatric version is supplemented later. Common used triage scales with specific parts for children are the Australasian Triage Scale (ATS), the Emergency Severity Index (ESI), the Manchester Triage System (MTS), and the Pediatric Canadian Triage and Acuity Score (paedCTAS) [6]. Most children are not taken to the specialty pediatric hospitals for the first visit, so nurses in general hospitals must make reliable decisions in all sort of situations for children presenting to emergency departments [1].

Several studies tried to shed light on the reliability of PTSs in the ED. van Veen provided an overview of the literature on reliability triage systems in pediatric emergency care and concluded that the MTS and paedCTAS both seem appropriate to triage children [6]. They reported that reliability of the triage scales is moderate at least, meaning that the reliability is good for the MTS, is moderate to good for the ESI, moderate for the paedCTAS, and poor to moderate for the ATS. They did not identify any triage scales superior to other scales in the ED.

Information is lacking on concordance between clinicians in terms of PTSs. Computing a pooled estimate of a reliability coefficient could help identify the potential effect of triage scales on triage concordance. Therefore, the aim of this meta-analytic review was to determine the level of agreement of PTSs in the ED.

## 2. Methods

### 2.1. Literature Search

**Figure 1:**
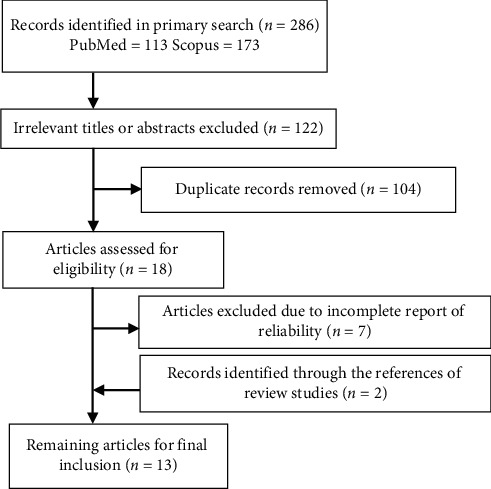
Flowchart of literature search and selection process.

Electronic databases, including Scopus and PubMed, were searched from conception to 1 March 2019. A publication date filter was not used. The search terms included “Reliability,” “Agreement,” “pediatric^*∗*^,” “child^*∗*^,” “Triage,” and “Emergency.” Relevant citations in the reference lists of the included studies were hand searched to identify other potential articles. Two researchers independently examined the search results to recover potentially eligible articles (Figure 1). If needed, the authors of the research papers were contacted to retrieve supplementary information.

### 2.2. Eligibility Criteria

Original full-text published studies were considered for inclusion. Studies were eligible if studies (1) used formal pediatric triage scales and (2) were of English-language publications. Formal triage scales included the Australasian Triage Scale (ATS), the Emergency Severity Index (ESI), the Manchester Triage System (MTS), and the Pediatric Canadian Triage and Acuity Score (paedCTAS). Irrelevant and duplicated results were eliminated.

### 2.3. Article Selection

Results of database searches were imported into Endnote X8.1 (Clarivate Analytics, Philadelphia, USA). Two independent reviewers (Z.N. and M.H.R.) assessed titles and abstracts for eligibility. Discrepancies were discussed to reach consensus, with unresolved cases taken to a third reviewer (M.E.).

### 2.4. Data Extraction

One reviewer (H.S.) used a predefined spreadsheet to extract study characteristics and measurement property data, including triage scale (type), participants (size), raters (profession and size), instruments (live vs. scenario), origin and publication year of the study, type of reliability coefficient (weighted vs. unweighted kappa), and reliability method (inter vs intra). The reliability coefficients extracted from the articles were as follows: inter-rater reliability, kappa coefficient (weighted and unweighted), intraclass correlation coefficient, Pearson's correlation coefficient, and Spearman's rank correlation coefficient. In a metaregression, each sample was considered a unit of analysis. If the same sample was reported in two or more articles, it was included once. In contrast, if several samples of different populations were reported in one study, each sample was separately included as a unit of analysis.

### 2.5. Evaluation of Extracted Data

Extracted data were evaluated according to the COnsensus-based Standards for the selection of health Measurement INstruments (COSMIN) taxonomy [8]: reliability (inter-rater reliability), internal consistency, measurement error, validity (criterion validity, content validity, construct), and responsiveness (Table 1). Internal consistency, test-retest reliability, and measurement error were not assessed further because no studies have used them to report reliability. Only studies that reported descriptions of the sample size, number of raters and subjects, sampling method, rating process, statistical analysis, and reliability coefficients were included in the analysis. Each of these items was graded qualified if described in sufficient detail in the paper. Because the aim of study focuses on reliability, validity and responsiveness were not assessed either. And interpretability and feasibility were assessed.

### 2.6. Data Analysis

Pooling data of reliability coefficients were performed for both types of reliability. The most qualified articles reported reliability coefficients using kappa statistics (an *r* type of coefficient, ranging from −1.00 to + 1.00). Kappa can be treated as a correlation coefficient in a meta-analysis. *Q* and *I*^2^ statistics were used to assess statistical heterogeneity among the selected studies. Variables with *Q* statistics with a *p* value < 0.05 were considered heterogeneous. It means that the amount of total variance was more than we would expect based on the within-study error, and hence a random-effects model was assumed [10]. To obtain the correct interpretation, back-transformation (*z* to *r* transformation) of the pooled effect sizes to the level of primary coefficients was performed [11, 12]. Fixed-effects and random-effects models were applied. The data were analyzed using the Comprehensive Meta-Analysis software version 2.2.050 (Biostat, Inc., Englewood, New Jersey, USA).

A simple metaregression analysis was performed according to the method of moments estimator [13]. In the metaregression model, to detect potential predictors of reliability coefficients, the effect size was considered the dependent variable, and the studies and characteristics of the subjects were considered the independent variables. The *z*-transformed reliability coefficients were regressed on the publication year of the study and distance. The latest version of the triage scale was identified through papers published after 2009 for the ATS, 2008 for the CTAS, 2014 for the MTS, and 2012 for the ESI. Distance was defined as the distance from the origin of each study to the place of origin for the triage scales. Melbourne (Australia), Boston (United State), Manchester (United Kingdom), and Edmonton (Canada) are considered as the origin of ATS, ESI, MTS, and CTAS triage scales, respectively. The metaregression analysis was performed using a random-effects model because of the presence of significant between-study variation [14].

### 2.7. Quality Assessment

The COSMIN checklist with four-point rating scale (excellent, good, fair, and poor) was used to assess methodological quality of included studies. Two reviewers (A.M. and M.E.) independently rated each study. Disagreements were discussed to reach consensus. Overall ratings of methodological quality were based on COSMIN guidelines. Since pooled coefficients were generally not different between COSMIN ratings, all studies were included in the meta-analyses.

## 3. Results

### 3.1. Study Selection and Characteristics

The search strategy identified 286 primary citations relevant to the present study on the agreement of the pediatrics triage system in the ED. A total of 13 articles met the inclusion criteria (Figure 1). Subgroups were organized for triage scales (ATS, CTAS, ESI, and MTS), raters (expert, physician and nurse), and reliability statistics (weighted and unweighted *κ*). The level of agreement among the reviewers in the final selection of the articles was almost perfect (*κ* = 1.0). The total sample size from all studies was 29,094. The studies were conducted in five countries (Australia, Canada, Iran, Netherlands, and the USA) (Table 2). The publication year of the studies ranged from 2002 to 2015 (median: 2009). Seventy percent of all the studies were conducted using the latest version of the triage scale. All the studies used inter-rater reliability. The weighted *κ* coefficient was the most commonly used statistic (Table 2). The overall pooled coefficient was 0.677, denoting substantial agreement of PTSs (95% confidence interval (CI): 0.671–0.683; *z* value: 140.32, *p* < 0.001, Q-value model: 3376.12, df: 24, *p*=0.001; *I*^2^ = 99%; Tau^2^ = 0.127) for the fixed-effects model and 0.723 (95% (CI): 0.648–0.784; *z* value: 12.658, *p* < 0.001) for the random-effects model.

### 3.2. Subgroup Analyses

**Figure 2:**
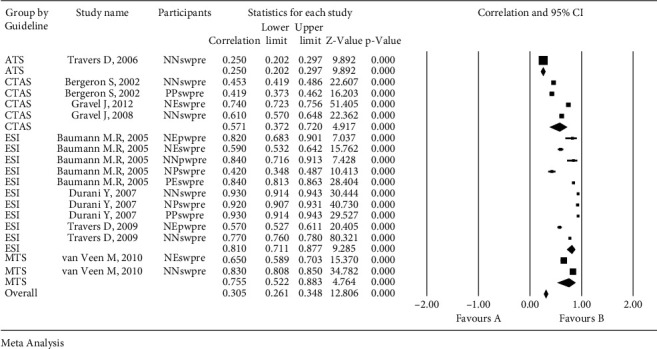
The pooled coefficient estimates of triage reliability coefficients.

The level of agreement on PTSs (based on weighted *κ* statistic) ranged from slight to almost perfect, with a value of 0.25 (95% CI: 0.202–0.297) for ATS, 0.571 (95% CI: 0.372–0.720) for CTAS, 0.810 (0.711–0.877) for ESI, and 0.755 (95% CI: 0.522–0.883) for MTS (Figure 2 and Table 3). The weighted *κ* and unweighted *κ* was 0.736 and 0.689, respectively, denoting substantial reliability in the level of agreement of PTSs (95% CI: 0.644–0.807 and 0.567–0.781), respectively. The level of agreement among raters based on weighted *κ* statistics was generally substantial, with a value of 0.747 (95% CI: 0.546–0.866) for nurse-nurse, 0.769 (95% CI: 0.100–0.973) for nurse-physician, 0.659 (95% CI: 0.574–0.729) for nurse-expert, 0.782 (95% CI: 0.35–0.978) for physician-physician, and 0.840 (95% CI: 0.813–0.863) for physician-expert raters. Furthermore, the level of agreement of PTSs in terms of their assessments of actual patients and paper-based scenarios (based on weighted *κ* statistic) were also substantial, with values of 0.709 (95% CI: 0.609–0.786) and 0.740 (95% CI: 0.608–0.832), respectively.

### 3.3. Moderator Effect

A metaregression analysis was performed based on the method of moments for distance and publication year. Studies reported a pooled coefficient for locations closer to the origin of the triage scales differ significantly from the origin of the PTSs, CTAS, and ESI (Table 4). The regression coefficient for PTS slope is 0.0005, which means that every one kilometer of distance corresponds to an increase of 0.0005 units in effect size. It indicates that PTSs are generalizable to the other countries. Also, the reliability of PTSs improved over time.

### 3.4. Sensitivity Analysis

Egger's regression intercept test demonstrated that the asymmetry of funnel plot is not statistically significant (*p* value = 0.51). Two subgroups of Baumann's study showed a standard error of 0.164 [15]. We did not exclude them from the final analysis because a symmetrical funnel plot was used. Also, a sensitivity analysis showed that two studies played a significant role in increasing the agreement level of pediatric ESI triage scale from 0.672 (0.642–0.701) to 00.787 (0.710–0.845) based on unweighted *κ* statistics [23, 24].

### 3.5. Interpretability and Feasibility

Triage scales are highly interpretable because they use 5-level ranging from the highest acuity level (1) to the lowest acuity level (5). Triage scales are feasible, and no issue is reported.

## 4. Discussion

The results revealed a substantial level of agreement for pediatric triage systems. Thus, the inter-rater reliability of triage scales appears to be acceptable. As a result, triage decisions are consistent in the pediatric triage decision-making. The level of agreement on PTSs ranged from 0.250 to 0.810. The ATS showed slight agreement of 0.309. But we should keep in mind that only three Australian studies were available for the assessment of ATS's reliability [3, 17]. More research is needed to reach a final conclusion about the ATS's reliability. The paedCTAS showed moderate agreement of 0.571. All three studies were conducted in Canada, so the generalizability is limited to the Canadian EDs [16, 20, 22]. The ESI and MTS showed almost a perfect and substantial agreement level of 0.822 and 0.785, respectively. The reliability of ESI was evaluated by five studies, and three of them used unweighted *κ* statistics to report the level of agreement [15, 19, 23–25]. In contrast, the reliability of MTS was evaluated by only one study and two subgroups which used weighted *κ* statistics [26]. The MTS studies were exclusively conducted in the Netherlands. Since the level of agreement of ESI is higher than MTS, the ESI provides more robust reliability than any other triage scales. The ESI has simple structure and objective criteria. It is easy to learn and easily adaptable to other culture of care. More research studies are needed because only the reliability of ESI was evaluated by several studies and evidence is limited for other triage scales. Therefore, these findings are opposed to those of the van Veen et al. [6]. They concluded that the MTS and paedCTAS both seem valid to triage children. They reported that reliability of the triage scales is moderate at least, meaning that the reliability is good for the MTS, is moderate to good for the ESI, moderate for the paedCTAS, and poor to moderate for the ATS. The reason that lies behind this controversy refers to two recent studies that contributed to a notable increase in the reliability of the ESI triage scale from 0.672 to 0.787, which were performed after the van Veen et al. study.

Most of the studies (7 of 11) used weighted *κ* statistics to report agreement of PTSs [3, 15, 16, 19, 22, 25, 26]. Weighted *κ* statistics overestimate inter-rater reliability among raters. Weighted *κ* statistics demonstrate higher reliability than do unweighted *κ* statistics because they place more emphasis on large differences between ratings than that of small differences [27]. Small differences resulting in one category misclassification may endanger the life of critically ill patients. Therefore, unweighted kappa statistics provide a reasonable estimation of reliability in terms of the level of agreement of PTSs. In the present study, the weighted *κ* and unweighted *κ* were 0.743 and 0.723, respectively, denoting substantial reliability in the level of agreement of PTSs. Six studies used unweighted *κ* statistics to report the level of agreement [17, 19, 20, 22–24]. Three of them were the studies that used ESI triage scale [23–25]. Two ESI studies played a significant role in increasing the agreement level of the pediatric ESI triage scale from 0.672 (0.642–0.701) to 0.787 (0.710–0.845) [23, 24]. However, it still remained at the substantial level of reliability, and these two studies notably increased the pooled coefficient of reliability. Both studies used the latest version of the ESI triage scale (version 4) [28]. It shows to what extent the latest version of the ESI triage scale may contribute to an increase in the level of reliability. More research studies are needed to confirm and explain this finding.

Weighted *κ* varied from moderate to almost perfect among raters (from 0.671 to 0.840). Physician-expert raters showed almost a perfect level of agreement. However, only one study reported the level of agreement between physician-expert raters, and this finding may be due to the fact that experts are usually responsible for the research and select physicians who are very similar to them in terms of expertise and experience in the ED. Physician-physician raters and nurse-nurse raters showed the reliability of 0.782 and 0.747, respectively. There may be several reasons contributing to the substantial level of agreement. However, nurses may not be knowledgeable of all aspect of emergency medicine as much as physicians, nurses primarily perform triage in the ED, so it is probable that their decision is affected by the practice effect and produces substantial agreement as much as that of physicians. In addition, physicians usually learn triage skills for research purposes and it may not result in almost the perfect level of agreement. Therefore, they rarely take part in the reliability research of triage scales which use flowchart diagrams such as MTS [6]. Based on our literature search, no studies seem to have assessed the level of agreement between physicians and nurses in MTS and ATS. The absence of studies on the inter-rater reliability of MTS between physicians and nurses is questionable. The latter may be due to the fact that the MTS is composed of 50 algorithms, and it is relatively complicated than the ESI. Thus, it is not rational for emergency physicians to learn the MTS only for research purposes. This may be true for the ATS as well. In contrast, it is easy to learn the ESI triage scale. Thus, most studies that assessed the level of agreement between emergency physicians and nurses have focused on the ESI. Five out of six studies in which physicians used as raters belong to the ESI triage scale because of simplicity embedded in the structure of ESI triage scale. All studies except one reported the level of agreement of nurse-nurse raters [22]. The level of agreement for nurse-nurse raters is substantial (0.747), which is in the range of overall agreement of PTSs (0.619). It is worth mentioning that nearly 75% of sample size involved nurse raters and 50% of sample size exclusively involved nurse-nurse raters. Of note, reliability differs from validity. Thus, substantial reliability does not necessarily indicate acceptable validity.

In the present study, the level of agreement of pediatric triage systems in terms of actual patients and paper-case scenarios was not congruent with that of the study by Worster et al. [29], and the use of actual patients was not associated with increased reliability as compared with that of paper-case scenarios (0.740 vs. 0.740). Worster et al. demonstrated that there was substantial to almost perfect agreement between actual patients and paper-case scenarios (0.9 vs. 0.76), with paper-case scenarios generally receiving lower triage scores than actual patients. Actual patients usually provide important clinical clues for raters, resulting in higher reliability of raters' triage scores. The reason is that we only included studies in which weighted kappa statistics was used. All studies that used actual patients to report agreement had been performed before 2012. Older version of PTSs may contribute to this effect.

As shown in Table 4, the concordance among raters has increased with time. This may due to multiple revisions of triage scales in recent years, supporting the idea that triage systems need to be regularly updated. The most increase is associated with the latest version of ESI, which increased the reliability of ESI from 0.672 to 0.789. In addition, raters have amassed a large amount of experience in the use of these scales since their introduction some years ago. They have also received training in the use of these scales in the ED.

The metaregression showed that triage scales are able to provide acceptable reliability in countries that are not the origin of triage scales. However, few studies were conducted outside of the origin of triage scales, and the ESI showed high reliability even in countries far from the origin of ESI [4]. In fact, the reliability coefficient in countries that are far from the country of origin of the scale was not lower than that of countries close to the origin of the triage scale. This may be due to the fact that the ESI framework is mostly objective and based on vital signs criteria [28]. The simplicity and objectivity of the ESI may limit the role of contextual and local factors in the reliability between physicians and nurses. This finding is congruent with that of a previous study on the reliability of ESI [4]. The ESI triage scale has been adapted for use in other countries, despite cultural diversities. However, triage systems should be compatible with culture of care in emergency department; more research studies are needed because no study has evaluated the reliability of MTS in the United Kingdom, and no study has evaluated the reliability of ATS and CTAS outside of Australia and Canada in pediatric triage systems, respectively [30].

The present study has a number of limitations. In the present analysis, more than half of studies used weighted kappa for inter-rater agreement between raters [3, 16, 19, 22, 25, 26]. As this study was partially limited to weighted kappa statistics, the results should be interpreted with caution, and potential overestimation of the pooled coefficients must be considered. However, subgroup analyses decreased this effect as much as possible. Contingency tables were not adequately reported in the included studies. Thus, we do not know whether the reliability differences among raters are related to which level of the PTSs. Most disagreement may possibly relate to triage levels 2–4, as patients in levels 1 and 5 are easily distinguishable from those in the other triage categories (levels 2–4). Patients in level 1 are clearly critically ill, and patients in level 5 are completely stable and ambulatory. A recent review on performance of triage scales indicated that most included studies had a risk of bias in at least one domain [31]. Therefore, they concluded that they cannot rule out that study design and methodological quality have led to heterogeneity of the results. Our review may suffer from extreme heterogeneity as well, so the findings must be interpreted with extreme caution [32].

Although the triage scale supports evidence-based practice in the ED [5, 6], there is a considerable gap between research and clinical practice, even at the best of times [27]. Therefore, the reliability in the level of agreement of PTSs in the field may differ from that reported herein. It is worth mentioning that triage reliability hardly exceeds the substantial level, except in situations where there are highly homogenous raters, such as expert-expert agreement or physician-expert agreement. In such cases, the reliability coefficient could be almost perfect (0.84).

## 5. Conclusion

Overall, the level of agreement of PTSs was substantial, and this level of agreement should be considered acceptable for triage in the ED. However, the Manchester Triage Scale and the Canadian Triage and Acuity Scale both seem valid to triage children; the reliability of Emergency Severity Index showed more robust reliability than any other pediatric triage scales. It does not necessarily mean that the ESI is more reliable than others because more studies with good quality are needed. The reliability of triage decisions has improved over time because of the practice effect and update on triage scales. Therefore, the reliability of PTSs needs more development to reach almost perfect agreement. Further studies on the level of agreement of PTSs are needed.

## Figures and Tables

**Table 1:** Quality criteria for evaluated measurement properties.

Measurement property	Definition	Data management and interpretation
*Reliability*		
Inter-rater reliability	It is the degree of agreement among raters	The agreement was defined as poor (*κ* = 0.00–0.20), fair (*κ* = 0.21–0.40), moderate (*κ* = 0.41–0.60), substantial (*κ* = 0.61–0.80), or almost perfect (*κ* = 0.81–1.00) [9] 1 Were patients stable in the interim period on the construct to be measured? 2 Were the test conditions similar for the measurements? For example, type of administration, environment, and instructions 3 For ordinal scores: was a weighted kappa calculated? 4 Were there any other important flaws in the design or statistical methods of the study?
	Design requirements	
	Statistical methods	
	Other	

Interpretability		
	Degree to which qualitative or clinical meaning can be assigned to the PTS scores or change in scores	Narrative synthesis

*Feasibility*		
Time to complete	Reported time taken for participants to complete the PTS	Narrative synthesis

**Table 2:** Studies on the agreement of pediatric triage scales in hospital triage.

Study name	Sample size	Raters number	Scenario/Patient number	Coefficient	Triage scale	Raters	Patient	Reliability method	Statistics	CO	Reliability properties			
Allen et al. [3]	1503	167	9	0.25	ATS	NN	Scenario	INTER	Kw	Australia	NA	A	V	V

Baumann et al. [15]		2			ESI			INTER	Kw	USA	NA	A	V	V
	40	2	20	0.84		NN	Actual patients							
	40	2	20	0.82		NE	Actual patients							
	544	2	272	0.59		NE	Scenario							
	544	2	272	0.42		NP	Scenario							
	544		272	0.84		PE	Scenario							

Bergeron et al. [16]	2145	39	55	0.453	CTAS	NN	Scenario	INTER	Kw	Australia	NA	A	V	V
	1320	24	55	0.419		PP								

Considine et al. [17]	1132	161	7	0.40	ATS	NN	Scenario	INTER	Kuw	Australia	NA	A	D	V

Crellin Johnston [18]	736	92	8	0.21	ATS	NN	Scenario	INTER	Kuw	Australia	NA	A	D	V

Durani et al. [19]	340	20	17	0.93	ESI	NN	Scenario	INTER	Kw	USA	NA	A	V	V
	660	20	33	0.92		NP								
	320	20	16	0.93		PP								
	340	20	17	0.67	ESI	NN	Scenario	INTER	Kw	USA	NA	A	D	V
	660	20	33	0.67		NP								
	320	20	16	0.68		PP								

Gravel et al. [20]	972	18	54	0.51	CTAS	NN	Scenario	INTER	Kuw	Canada	NA	A	D	V

Gravel et al. [21]	998	2	499	0.61	CTAS	NE	Actual patients	INTER	Kw	Canada	A	A	D	V

Gravel et al. [22]	2928	2	1464	0.74	CTAS	NE	Actual patients	INTER	Kw	Canada	A	A	V	V
	2928	2	1464	0.59	CTAS	NE	Actual patients	INTER	Kuw	Canada	A	A	D	V

Green et al. [23]	200	2	100	0.92	ESI	NN	Actual patients	INTER	Kuw	USA	A	A	D	V
	300	3	100	0.78		NP								

Jafar-Rouhi et al. [24]	2208	16	1104	0.82	ESI	NP	Actual patients	INTER	Kuw	Iran	A	A	D	V
	240	12	20	0.84	ESI	NN	Scenario	INTER	Kuw	Iran	NA	A	D	V

Travers et al. [25]	6200	155	40	0.77	ESI	NN	Scenario	INTER	Kw	USA	NA	A	V	V
	996	2	498	0.57	ESI	NE	Actual patients	INTER	Kw	USA	A	A	V	V

Van Veen et al. [26]	860	43	20	0.83	MTS	NN	Scenario	INTER	Kw	Netherlands	NA	A	V	V
	396	2	198	0.65	MTS	NE	Scenario	INTER	Kw	Netherlands	NA	A	V	V

NN = nurse-nurse; NP = nurse-physician; NE = nurse-expert; PP = physician-physician; PE = physician-expert; Kw = weighted kappa; Kuw = unweighted kappa; INTER = inter-rater reliability; CO = country of origin; VG = very good; A = adequate; D = doubtful; I = inadequate; NA = not applicable.

**Table 3:** Pooled coefficients (CI) of pediatric triage systems based on statistics.

Triage scales	All	Only studies with weighted kappa statistics	Only studies with unweighted kappa statistics
ATS	0.309 (0.113–0.482)	0.250 (0.202–0.297)	0.309 (0.113–0.482)
CTAS	0.554 (0.475–0.623)	0.571 (0.372–0.720)	0.553 (0.471–0.627)
ESI	0.822 (0.758–0.870)	0.810 (0.711–0.877)	0.787 (0.710–0.845)
MTS	0.755 (0.522–0.883)	0.755 (0.522–0.883)	–

**Table 4:** Metaregression of Fisher's *Z*-transformed *kappa* coefficients on predictor variables.

Independent variable	B	SEb	*p*
Distance from PTS origin^*∗*^	0.0005	0.00002	<0.001
Distance from ESI origin^*∗∗*^	0.0000	0.00003	<0.001
Distance from CTAS origin^*∗*^	0.0005	0.00003	<0.002
Publication year for PTSs	0.06	0.003	<0.001

SEb = standard error. ^*∗*^Only studies that used weighted kappa coefficients were included. ^*∗∗*^Only studies that used unweighted kappa coefficients were included.

## Abbreviations

ATS: Australasian Triage Scale
CI: Confidence interval
COSMIN: COnsensus-based standards for the selection of health Status Measurement INstruments
CO: Country of origin
CTAS: Canadian Triage and Acuity Scale
ED: Emergency department
ESI: Emergency Severity Index
Kw: Weighted kappa
Kuw: Unweighted kappa
INTER: Inter-rater reliability
MTS: Manchester Triage System
PTS: Pediatric triage scales

### Raters

NN: Nurse-nurse
NP: Nurse-physician
NE: Nurse-expert
PP: Physician-physician
PE: Physician-expert

### Reliability scoring

VG: Very good
A: Adequate
D: Doubtful
I: Inadequate
NA: Not applicable.

## Additional Points

*What is already known on this subject?* (i) The level of agreement on pediatric triage scales varied from poor to good. (ii) The Manchester Triage Scale and the Canadian Triage and Acuity Scale both seem valid to triage children. *What this study adds?* (i) The level of agreement on pediatric triage scales was substantial. (ii) However, the Manchester Triage Scale and the Canadian Triage and Acuity Scale both seem valid to triage children and the reliability of Emergency Severity Index showed more robust reliability than any other pediatric triage scales.
